# MRSLpred—a hybrid approach for predicting multi-label subcellular localization of mRNA at the genome scale

**DOI:** 10.3389/fbinf.2024.1341479

**Published:** 2024-02-06

**Authors:** Shubham Choudhury, Nisha Bajiya, Sumeet Patiyal, Gajendra P. S. Raghava

**Affiliations:** Department of Computational Biology, Indraprastha Institute of Information Technology, New Delhi, India

**Keywords:** subcellular localization, multi-label, motif search, messenger RNA, machine learning

## Abstract

In the past, several methods have been developed for predicting the single-label subcellular localization of messenger RNA (mRNA). However, only limited methods are designed to predict the multi-label subcellular localization of mRNA. Furthermore, the existing methods are slow and cannot be implemented at a transcriptome scale. In this study, a fast and reliable method has been developed for predicting the multi-label subcellular localization of mRNA that can be implemented at a genome scale. Machine learning-based methods have been developed using mRNA sequence composition, where the XGBoost-based classifier achieved an average area under the receiver operator characteristic (AUROC) of 0.709 (0.668–0.732). In addition to alignment-free methods, we developed alignment-based methods using motif search techniques. Finally, a hybrid technique that combines the XGBoost model and the motif-based approach has been developed, achieving an average AUROC of 0.742 (0.708–0.816). Our method—MRSLpred—outperforms the existing state-of-the-art classifier in terms of performance and computation efficiency. A publicly accessible webserver and a standalone tool have been developed to facilitate researchers (webserver: https://webs.iiitd.edu.in/raghava/mrslpred/).

## 1 Introduction

Messenger RNA (mRNA) is a single-stranded RNA, a transcription product that leads to protein synthesis via translation. It carries the cell’s genetic information from the nucleus to the cytoplasm. In the cytoplasm, mRNA is localized to different parts of the cell, resulting in an asymmetric distribution of proteins within the cell (Wang et al., 2021). It plays an important role in several developmental processes, such as neuronal maturation, embryonic patterning, cell migration, cell fate determination, cell adaptation to stress, and the development of body axes in *Drosophila melanogaster* (Ephrussi et al., 1991; Katz et al., 2012; Medioni et al., 2012; Liu et al., 2019; Tian et al., 2020). Identifying the cellular location of mRNA provides valuable information about the amount of protein synthesis and the location, which correlates with its function (Holt and Bullock, 2009; Tang et al., 2021). Transporting mRNA over a protein has significant advantages, such as transportation cost reduction by the expression of mRNA to generate different types of localized proteins, rapid response to external stimuli, segregation of transcripts to specific organelles or compartments, and prevention of ectopic action of proteins during localization (Kloc et al., 2002; Di Liegro et al., 2014; Tang et al., 2021; Wang et al., 2021). Therefore, knowing the subcellular localization of mRNA is important to understand various biological processes (Tang et al., 2021).

In the past, numerous experimental techniques have been established to identify the location of mRNA in a cell. *In vitro* visualization can be performed using classical *in situ* hybridization, MS2-system-based techniques, and RNA (Fagerberg et al., 2014; Wang et al., 2021; Zhang et al., 2021). These experimental techniques are highly sensitive and accurate, capable of detecting subcellular localization with high precision. Despite their accuracy, these techniques cannot be implemented routinely at the genome level due to their cost. Experimental techniques are laborious, have limited applications to specific tissues, are expensive processes, and often require sophisticated instrumentation (Savulescu et al., 2021). Advanced sequencing techniques generate a large amount of information about transcripts. To overcome these challenges, researchers have developed a wide range of *in silico* methods for predicting the location of mRNA in a cell (Savulescu et al., 2021). Most of these *in silico* methods are knowledge-based, deriving rules or models from experimental data to predict the location of an mRNA sequence. One can obtain experimental data from major repositories such as RNALocate (Zhang et al., 2017; Cui et al., 2022), lncATLAS (Mas-Ponte et al., 2017), and lncSLdb (Wen et al., 2018).

Over the years, a multitude of methods have been developed for predicting subcellular localization, with the majority relying on machine learning techniques. Examples include RNATracker (Yan et al., 2019), iLoc-mRNA (Zhang et al., 2021), DM3Loc (Wang et al., 2021), mRNALocater (Tang et al., 2021), and mRNALoc (Garg et al., 2020). In most of these methods, datasets have been derived from the popular RNALocate database. One major limitation of these tools is their tendency to predict or assign a single label for a given mRNA. However, in reality, most mRNAs traverse throughout the cell and can be found at multiple locations within it. Lin et al. developed a method named DM3Loc (Wang et al., 2021), specifically designed to predict multiple labels or locations for a given mRNA. This method, referred to as a multi-label subcellular localization prediction method, is based on a deep learning framework. DM3Loc generates features by converting mRNA sequences into one-hot encoded vectors. These vectors serve as input to a CNN classifier, and a multi-head self-attention mechanism is used to enhance its ability to identify sequence regions relevant to localization. Currently, DM3Loc stands as a state-of-the-art multi-label classifier for predicting the subcellular localization of mRNA sequences. Despite its advanced capabilities, DM3Loc has limitations of its own, such as heavy requirements for computational resources and time.

In this study, a novel multi-label classifier is proposed for predicting the subcellular localization of mRNA. This method, named MRSLpred, is based on machine learning and uses the composition features of mRNA sequences for prediction. Importantly, MRSLpred can be implemented at a genome scale as its computational resource requirements are minimal. This proposed method aims to complement existing approaches and address some of the limitations associated with current methods.

## 2 Materials and methods

### 2.1 Dataset collection

**FIGURE 1:**
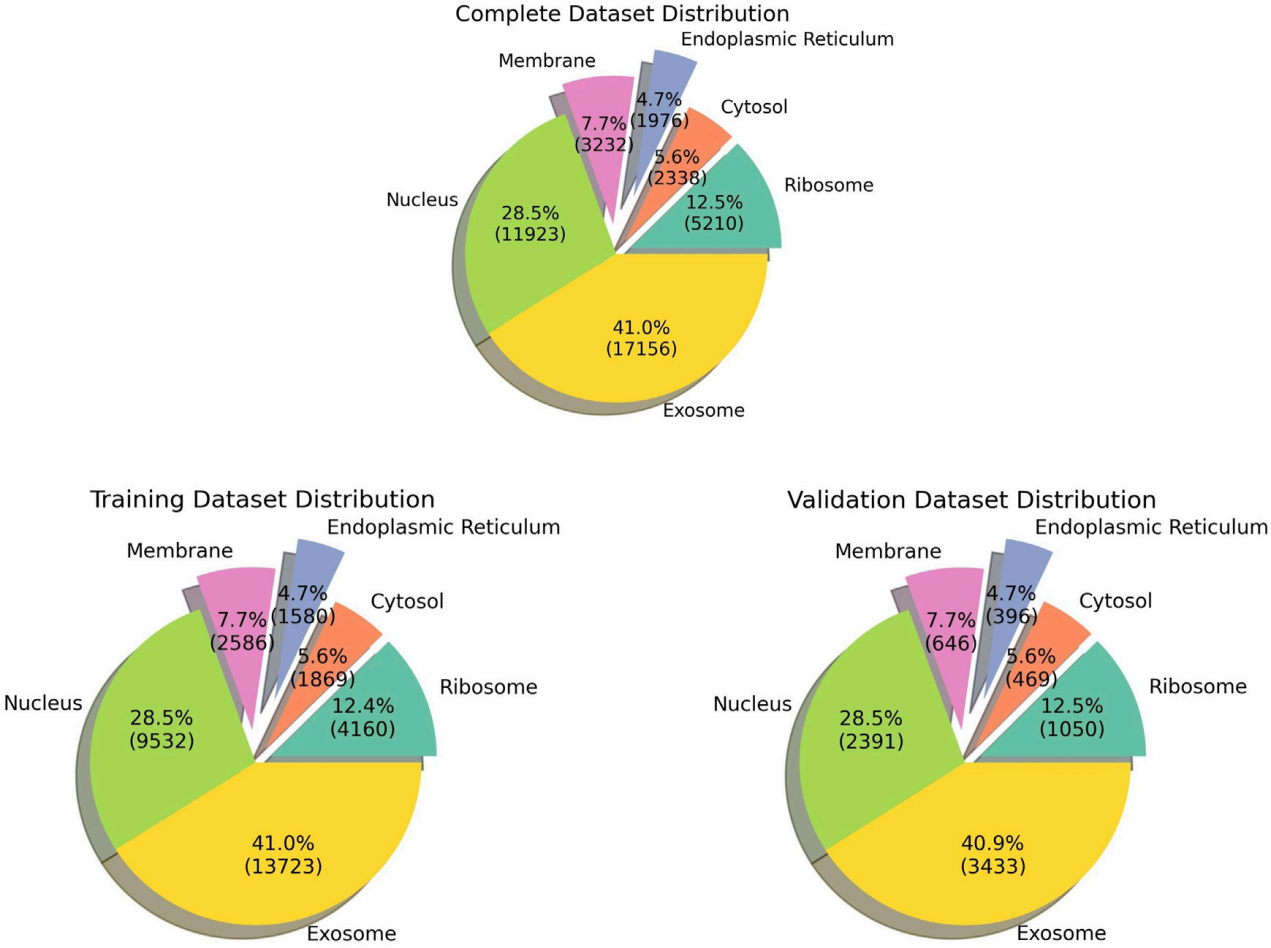
Pie chart indicating the data distribution in all the datasets.

**FIGURE 2:**
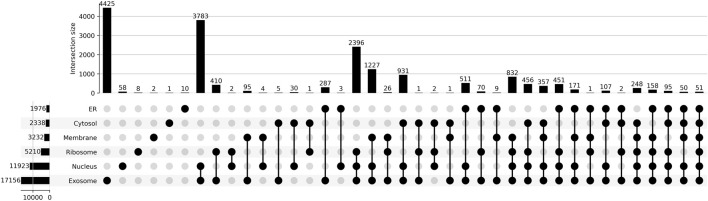
Upset plot depicting all the possible combinations of subcellular locations.

The pre-processed dataset was obtained from DM3Loc (Wang et al., 2021), where they have used both experimentally validated and database-curated mRNA sequences of *Homo sapiens* from RNALocate database v2 (Cui et al., 2022). RNALocate is a database dedicated to providing high-confidence RNA subcellular localization information sourced from the literature, other databases, and RNA-seq datasets. A majority of the mRNAs were localized in more than one subcellular compartment, which is generally the case with most mRNAs in a real-world scenario. In order to ensure that the dataset is non-redundant, CD-HIT was run at a threshold of 80% similarity. Our dataset contains a total of 17,277 non-*exclusive* human mRNAs spread across six subcellular compartments: the ribosome, cytosol, endoplasmic reticulum (ER), membrane, nucleus, and exosome. The dataset is graphically represented in Figure 1. The number of mRNAs with localization at different compartments is 11,923 for the nucleus, 17,156 for the exosome, 2,338 for the cytosol, 5,210 for the ribosome, 3,232 for the membrane, and 1976 for the ER. The distribution of location labels is depicted in Figure 2.

### 2.2 Feature generation—composition-based features for machine learning models

In order to train our model, we are required to generate features or descriptors corresponding to each mRNA. For the aforementioned purpose, we used the tool ‘Nfeature’ (Mathur et al., 2021) [https://doi.org/10.1101/2021.12.14.472723], which can generate hundreds of features for a single mRNA sequence. These are the two feature classes which were used for training the models:

1. Composition of DNA/RNA for k-mer (CDK): k-mers of length 3 were generated using Nfeature, and the frequency of each k-mer was used as a feature for training the ML model. It was calculated using the following formula:

$$CDK_{i}=\frac{N_{i}}{L-i+1},$$

where $N_{i}$ represents the number of occurrences of the k-mer *i* in the mRNA sequence and *L* represents the length of the mRNA. For example, if ‘ATG’ is a 3-mer, the program will count all the instances of this 3-mer in the input sequence and divide it by the length of the input sequence minus 2.

2. Reverse complement of DNA for k-mers (RDK): k-mers of length 4 were generated using Nfeature, and the frequency of the reverse complement of this k-mer will be used as a feature. The formula used for calculating the RDK is similar to the one used for the CDK. However, in this case, instead of counting the occurrence of the k-mer in the input sequence directly, first, the reverse complement of the sequence is generated, and then, the 4-mer frequency in that sequence is calculated.

Both these features were combined together to obtain a vector of 200 features for each mRNA.

### 2.3 Alignment-based methods—motif search using MERCI

Motifs are known to play a significant role in the localization of mRNA within the cell. We used the MERCI tool (Vens et al., 2011) to identify the presence of conserved motifs in the training dataset. For each location, the dataset was split into a positive and a negative dataset based on the localization in that location. Both the positive and negative datasets are then provided as inputs to MERCI (Vens et al., 2011), and then, the tool identifies motifs that can discriminate between positive and negative samples for that location. We acquired six sets of motifs specific to each location, and this information was used to modify the prediction probabilities for each location. For instance, in case a motif specific to ribosome sequences is found within a query sequence, the probability (provided by the ML model) that the query sequence belongs to the ribosome is updated to 1. So, the presence of motifs will practically override the prediction made by the ML model, and those sequences that do not contain any motifs remain unaffected.

### 2.4 Alignment-free methods—machine learning and deep learning models

The location label of each mRNA was generated using one-hot encoding by converting locations into 0 or 1 s; i.e., if an mRNA is only present in the ribosome and the nucleus, it will have a label like [1,0,0,0,1,0]. Initially, a CNN model was trained using one-hot encoded mRNA sequences. One-hot encoding was performed by converting every mRNA sequence into a 2000×4 matrix, where the columns represent the four nucleotides (A, T, C, and G) and the rows represent the sequence information. For sequences greater than 2000 base pairs (bp), 1,000 bp were taken from the 5′-end, and the remaining 1,000 bp were taken from the 3′-end. Sequences that have less than 2000 bp were used as they are, and the remaining matrix was filled with zeros for all the remaining positions.

Composition-based features defined above were used to train various machine learning models. Model training was conducted on Python using standard machine learning libraries such as *scikit-learn* and *XGBoost* (Chen and Guestrin, 2016). A number of machine learning approaches were used to construct prediction models such as logistic regression, decision tree, random forest classifier, MLP classifier, AdaBoost classifier, Gaussian Naïve Bayes, quadratic discriminant analysis, gradient boosting classifier, and eXtreme Gradient Boosting (XGBoost) classifier. The *scikit-learn* Python library was used to implement these classification approaches.

Training of the models was conducted using ML classifiers combined with a multioutput classifier. The specialty of this model is that it takes CDK and RDK as features and predicts all the possible locations of the mRNA in one single step.

### 2.5 Five-fold cross validation

To ensure the proper model fitting, we used five-fold cross-validation to train and validate the models. The entire dataset was split in an 80:20 ratio, where 80% of the data were used for training and 20% data were used for validation. The training data were further split into five parts, and five-fold cross-validation was performed on the same. In each iteration, a different fold was used for validation, and the remaining four folds were used for validation. The training performance is calculated by taking the average over five iterations. The splitting of data was performed in a stratified manner, ensuring that all the locations were equally distributed within each of the folds. Once the training was conducted, the model was validated using the 20% validation dataset.

### 2.6 Performance metrics

Evaluation of the model performance was carried out using standard performance metrics. The performance metrics used were sensitivity, specificity, accuracy, Matthews’ correlation coefficient, F1-score, and area under the receiver operator characteristic (AUROC). Out of these metrics, only the AUROC is threshold-independent, whereas all the remaining metrics are dependent on the threshold cut-off. The cut-off for the probabilities was determined by balancing out the sensitivity and specificity as follows:

$$Sensitivity=\frac{TP}{TP+FN},$$

$$Specificity=\frac{TN}{TN+FP},$$

$$Accuracy=\frac{TP+TN}{TP+TN+FN+FP},$$

$$F1-score=\frac{TP}{TP+\left(0.5\times \left(FN+FP\right)\right)},$$

$$MCC=\frac{\left(TP\times TN\;\right)-\;\left(FP\times FN\right)}{\sqrt{\left(TP+FP\right)\times \left(TP+FN\right)\times \left(TN+FP\right)\times \left(TN+FN\right)}},$$

where TP is true positive, FP is false positive, FN is false negative, and TN is true negative.

## 3 Results

**FIGURE 3:**
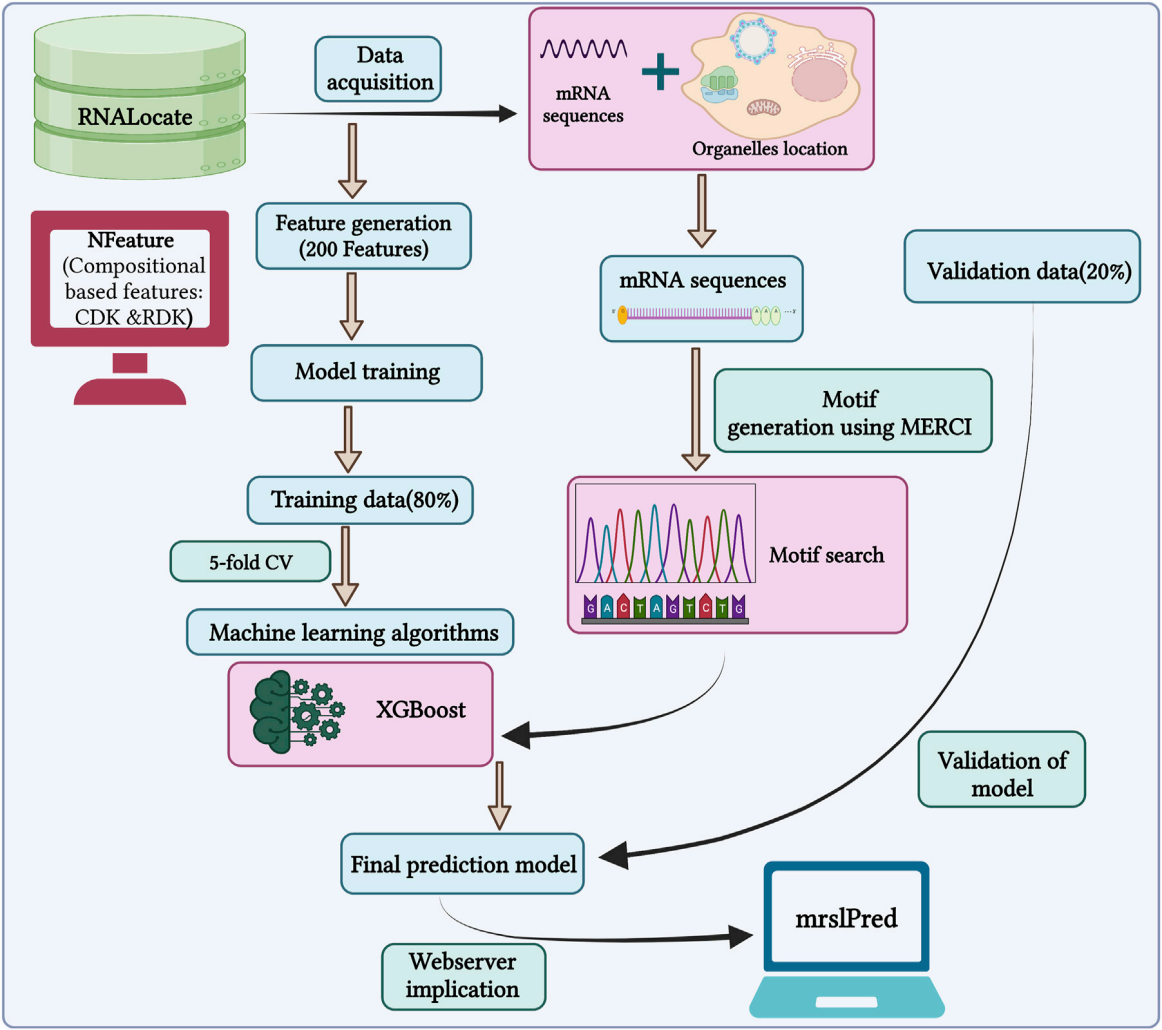
Outline of the methodology followed by MRSLpred.

In this study, we used a total of 17,277 mRNA sequences with non-exclusive locations for training our model. The primary objective is to develop a model that can accurately predict multiple locations for a single mRNA, mimicking a practical scenario within the cell. So, we developed a multi-label classifier to predict the subcellular localization of mRNA. The outline of the study is shown in Figure 3.

### 3.1 Alignment-based method—the motif search module

Nucleotide motifs are known to affect mRNA localization, and in this study, we tried to implement this ideology. Discriminatory motifs for each location were identified using the training dataset, and the presence of these motifs was then used to assign location labels to each mRNA in the validation dataset. The motifs that are unique to the positive dataset for each location are searched in the validation dataset, and if any one of these motifs is found within the sequence, then the label for that mRNA in that location is assigned as 1 or 0. The number of hits for motifs in each individual location was as follows—ribosome: 33, cytosol: 25, endoplasmic reticulum: 66, membrane: 29, nucleus: 96, and exosome: 1,655. The top 10 motifs found in each location are provided in Table 1. A large number of motifs were predominantly identified in the mRNA sequences that were assigned to exosomes and nucleus. This may be possibly due to the relatively high number of sequences assigned to the exosome location. This can be seen in Table 2, where the number of unique motifs identified by MERCI in each location is provided, as well as the total number of hits for these motifs found in the training dataset.

**TABLE 1:** Sequence of top 10 motifs exclusively found in each subcellular location; all these motifs were discovered in the training dataset using MERCI software.

Ribosome motif (hits)	Cytosol motif (hits)	ER motif (hits)	Membrane motif (hits)	Nucleus motif (hits)	Exosome motif (hits)
ATTTGAAGACCA^a^ (5^b^)	CTG​CCA​CCA​CGC​CCA​GCT​AAT​TTT​TT (4)	CAGGGGAATGCA (3)	ATAGCAGTTTCT (3)	CTAAGAGAGT (15)	AATTTGTA (339)
GGTGTGGATGAG (4)	CCT​GCC​ACC​ACG​CCC​AGC​TAA​TTT​TTT (4)	GTGGTGATCCAGC (3)	ATCAGATCAGAA (3)	GTGGATTTAAA (13)	GCTGCAAA (316)
CAAAGGAATGAG (3)	CGCCCAGCTAATTTTTA (4)	CCACCTTCGAGA (3)	TAAGAAAAAAACT (3)	AGT​GCT​GGG​ATT​ACA​GGC​GTG​AGC​CAC​CAC (11)	TGGATTTA (313)
GCCAACAAAGAA (3)	AGA​CAG​GGT​CTC​ACT​CTG​TCA​CC (3)	CCACAGTAGAAT (3)	TATTATTTATAAA (3)	GTG​CTG​GGA​TTA​CAG​GCG​TGA​GCC​ACC​AC (11)	GAAGTTGA (304)
CATTGGATACT (3)	TCT​TGT​CGC​CCA​GGC​TGG​AGT​GCA​G (3)	AACCCCTTCGTG (3)	GTCCAGAAAATG (2)	CATTTTATGCA (11)	ATGAACTT (298)
CAGACAGGGCG (2)	GTGATCTCGGCTT (3)	GCACCCTGGACGA (2)	ACTGGCTGGATT (2)	CTGTTGAAGCA (9)	AATTGTAT (279)
CCAGCCTGGCCAACC (2)	GGCGTGATCTCGGCTT (3)	ACC​AGC​CTG​GAC​AAC​ATA​GTG (2)	GCAATTGAACC (2)	GCATTTTGTAT (8)	ACAGGAAC (275)
GTCATTTGTTCT (2)	GCGTGATCTCGGCTT (3)	GGCAAGGATGCTG (2)	AAATCTGGATGC (2)	CCCCTCCCCCCG (6)	TTTCAACT (274)
TTTTAAACCTTTTT (2)	CTT​GTC​GCC​CAG​GCT​GGA​GTG​CAG (3)	GCTGTGGCTGCTGCTG (2)	AGATGAAAATGAAG (2)	ATGTAAATTGT (6)	AATTACAG (263)
GCTATATTTCC (1)	CTC​TTG​TCG​CCC​AGG​CTG​GAG​TGC​AG (3)	ATTGCTTCATCTG (2)	CATTTTACAGGC (2)	TTGAAGCCAGGA (6)	TGCAGTCT (249)

^a^ Sequence of a motif.

^b^ Number of hits found in the dataset.

**TABLE 2:** Number of location-specific unique motifs found in the training dataset and the number of sequences in the training dataset containing these motifs (coverage).

Subcellular location	Number of unique motifs	Total number of sequences containing these motifs
Ribosome	16	32
Cytosol	32	25
ER	104	65
Membrane	14	29
Nucleus	13	95
Exosome	10	1,655

### 3.2 Performance of alignment-free methods—the machine learning model using composition-based features

Different composition-based features were used to train the ML model, and we achieved maximum average AUROCs of 0.705 and 0.691 and average MCCs of 0.213 and 0.195, respectively, in the cases of CDK and RDK. Upon the combination of these two features, the model performance further improved, giving an AUROC in the range of 0.709 and an MCC of 0.216.

Training of models was conducted using standard python packages—decision tree, random forest classifier, MLP classifier, AdaBoost classifier, Gaussian Naïve Bayes, quadratic discriminant analysis, gradient boosting classifier, and eXtreme Gradient Boosting classifier. The results for all the models are shown in Supplementary Table S1. Initially, composition-based features were used for training the models, and the XGBoost classifier had the best performance among all the ML models. The hyperparameters for the XGBoost model that reported the best performance were as follows: MultiOutputClassifier [XGBClassifier (n_estimators = 1,000, learning_rate = 0.01, random_state = 1, max_delta_step = 1, and n_jobs = −1)]. The metrics for the best performing model are shown in Table 3.

**TABLE 3:** Performance of the native XGBoost multi-label classifier on CDK3+RDK4 features.

Location	Sensitivity	Specificity	Accuracy	MCC	F1-score	AUROC
Ribosome	0.654	0.675	0.668	0.306	0.545	0.721
Cytosol	0.648	0.649	0.649	0.208	0.334	0.703
ER	0.641	0.607	0.611	0.16	0.274	0.668
Membrane	0.67	0.669	0.669	0.27	0.431	0.732
Nucleus	0.668	0.658	0.665	0.304	0.733	0.728
Exosome	0.546	0.731	0.548	0.048	0.706	0.706
Average	0.638	0.665	0.635	0.216	0.504	0.71

### 3.3 Final model—the XGBoost + motif module

The best performance was obtained by combining the alignment-free XGBoost model with the motif module. Once the ML model makes the prediction, the presence of these motifs was then used to tweak the prediction. If any motif is found in the query sequences, the prediction probability from the ML model is switched to 1. For instance, if a motif unique to the ribosome is found within a query sequence, and the prediction probability for that location by the ML model is 0.4, then in the final model, it will be assigned a probability of 1. Furthermore, if no motifs were found for the same query sequence, then the prediction probability assigned by the ML model remains unchanged.

**FIGURE 4:**
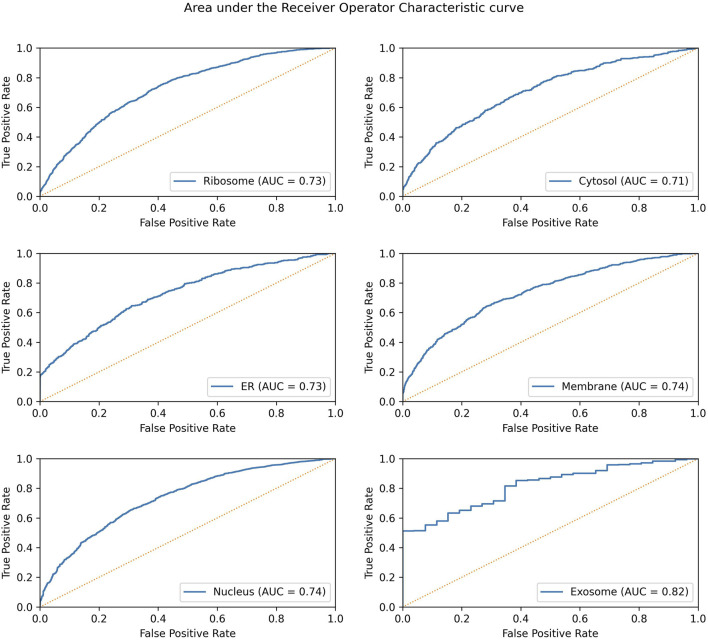
AUROC for the final model in each individual location. The AUROC is calculated on the validation dataset, and the yellow dotted line represents the AUROC for a random prediction (AUROC = 0.5).

Significant performance improvement was observed upon implementing this module. The performance metrics for the ML + motif module are provided in Table 4. The area under the receiver operator characteristic curve for each of the individual locations is shown in Figure 4.

**TABLE 4:** Performance of the XGBoost multi-label classifier combined with the motif module (final model).

Location	Sensitivity	Specificity	Accuracy	MCC	F1-score	AUROC
Ribosome	0.664	0.664	0.664	0.304	0.545	0.728
Cytosol	0.65	0.65	0.65	0.211	0.335	0.708
ER	0.657	0.656	0.656	0.205	0.304	0.727
Membrane	0.675	0.676	0.676	0.28	0.437	0.736
Nucleus	0.671	0.671	0.671	0.319	0.738	0.736
Exosome	0.696	0.692	0.696	0.073	0.82	0.816
Average	0.669	0.668	0.669	0.232	0.53	0.742

### 3.4 Comparison of MRSLpred with existing tools

Currently, DM3Loc is the only tool that performs multi-label classification out of the box and is developed on the latest version of RNALocate (version 2). We also used the same non-redundant dataset that is used in DM3Loc. However, the dataset splitting is performed differently, maintaining a similar proportion of subcellular locations in each split. The performance of both DM3Loc and MRSLpred was evaluated on the validation dataset, and it was observed that DM3Loc performs better than MRSLpred in terms of AUROC and MCC. However, the better performance of DM3Loc can be attributed to the bias in favor of DM3Loc as some of the sequences in the validation dataset are present within the training dataset used in DM3Loc. This leads to an overreported result when evaluating DM3Loc on our validation result.

Both MRSLpred and DM3Loc have been designed as multi-label classifiers where more than one location can be assigned to a single sequence. On the other hand, other existing methods perform multiclass classification, assigning only one location to a single sequence, and comparing MRSLpred/DM3Loc with these tools would be unfair to them. However, for the sake of comparison, we evaluated the performance of all these tools on the validation dataset used in MRSLpred. The detailed comparison of MRSLpred with DM3Loc and all other tools which do not support multi-label classification is provided in Table 5.

**TABLE 5:** Benchmarking of MRSLpred with existing prediction tools on the validation dataset used in MRSLpred.

Location	Sens	Spec	Prec	Acc	MCC	F1-score	AUROC
MRSLpred							
Ribosome	0.664	0.664	0.463	0.664	0.304	0.545	0.728
Cytosol	0.650	0.650	0.226	0.650	0.211	0.335	0.708
ER	0.657	0.656	0.198	0.656	0.205	0.304	0.727
Membrane	0.675	0.676	0.323	0.676	0.280	0.437	0.736
Nucleus	0.671	0.671	0.821	0.671	0.319	0.738	0.736
Exosome	0.696	0.692	0.997	0.696	0.073	0.820	0.816
DM3Loc							
Location	Sens	Spec	Prec	Acc	MCC	F1-score	AUROC
Ribosome	0.699	0.785	0.587	0.759	0.464	0.638	0.821
Cytosol	0.518	0.876	0.396	0.827	0.353	0.449	0.795
ER	0.351	0.934	0.409	0.868	0.305	0.378	0.789
Membrane	0.638	0.795	0.416	0.765	0.373	0.504	0.806
Nucleus	0.755	0.766	0.878	0.758	0.490	0.812	0.841
Exosome	0.785	0.885	0.999	0.786	0.140	0.879	0.894
mRNALoc							
Location	Sens	Spec	Prec	Acc	MCC	F1-score	AUROC
Cytosol	0.426	0.503	0.119	0.492	−0.049	0.186	0.464
Nucleus	0.244	0.647	0.612	0.367	−0.112	0.349	0.446
ER	0.172	0.756	0.084	0.690	−0.053	0.113	0.464
iLoc-mRNA							
Location	Sens	Spec	Prec	Acc	MCC	F1-score	AUROC
Ribosome	0.088	0.778	0.147	0.569	−0.160	0.110	0.433
Cytosol	0.119	0.809	0.089	0.716	−0.063	0.102	0.464
ER	0.535	0.865	0.339	0.827	0.331	0.415	0.700
Nucleus	0.152	0.754	0.580	0.338	−0.113	0.241	0.453
Exosome	0.180	0.692	0.987	0.184	−0.029	0.305	0.436

Sens, sensitivity; Spec, specificity; Acc, sccuracy; MCC, Matthews correlation coefficient; AUROC, area under the receiver operator characteristic curve.

Another significant advantage of MRSLpred is that it is computationally very efficient and consumes very less time. A time comparison between both the methods was performed on an iMac (21.5-inch, Late 2015 model, 2.8 GHz Quad-Core Intel Core i5, 8 GB DDR3 RAM, and Intel Iris Pro Graphics 6,200–536 MB). The detailed comparison of MRSLpred and DM3Loc based on the time taken to generate predictions is provided in Table 6.

**TABLE 6:** Comparison of MRSLpred with DM3Loc based on the time taken for similar datasets.

	Number of nucleotides	Real time	User time	System time
DM3Loc	20	0m47.609 s	1m13.874 s	0m2.402 s
	50	2m2.065 s	2m48.248 s	0m3.526 s
	100	3m28.185 s	5m28.421 s	0m6.338 s
	500	13m57.518 s	28m2.371 s	0m42.828 s
	Number of nucleotides	Real time	User time	System time
MRSLpred	20	0m13.659 s	0m3.999 s	0m0.950 s
	50	0m22.970 s	0m5.101 s	0m1.020 s
	100	0m24.121 s	0m6.965 s	0m0.971 s
	500	0m19.649s	0m20.237s	0m0.783s

## 4 Discussion

mRNA localization is a relevant biological process that controls the concentration of mRNA at different locations. This, in turn, exerts control on the level of protein translation at various locations within the cell (Kindler et al., 2005). Understanding mRNA subcellular localization will provide a better perspective on how protein synthesis is regulated at the mRNA level. Spatial localization of mRNA is also gathering massive interest in the field of development biology. The majority of developmental processes rely on cellular polarity generated by mRNA localization to undergo differentiation (Lécuyer et al., 2007; Claußen et al., 2015). A lot of research work is focused on understanding these spatial processes, which, when perturbed, can lead to major developmental disorders.

However, *in vitro* investigation of mRNA localization is a costly business and labor-intensive at the same time. Computational techniques can provide a viable solution to this problem, as they are swift, inexpensive, and reliable. Modern machine learning and deep learning techniques manage to perform well on biological data and are pretty accurate.

Many tools have already been developed for mRNA subcellular localization prediction. DM3Loc (Wang et al., 2021) is a popular tool that deploys a multi-head self-attention mechanism with a CNN model. DM3Loc uses a one-hot encoding vector as a feature for the CNN model and achieves an AUROC in the range of 0.698–0.773 (average: 0.742) and an MCC in the range of 0.074–0.386 (average: 0.270) on their own validation dataset. However, the performance of DM3Loc on our validation dataset painted a different picture. It only achieved an AUROC in the range of 0.557–0.776 (average: 0.704) and an MCC in the range of 0–0.353 (average: 0.081). As of now, only DM3Loc performs multi-label classification, whereas all the other tools are multiclass classifiers. Due to its complex architecture, DM3Loc requires very intensive computational power that includes a dedicated graphics card. For many sequences, one-hot encoding mRNA sequences is a herculean task, as most of the time, the RAM crashes due to the increasing size of the vector.

MRSLpred achieves an AUROC between 0.708 and 0.816 (average: 0.742) and an MCC between 0.073 and 0.319 (average: 0.232) on the validation dataset. MRSLpred uses a much simpler approach, combining an XGBoost model with a motif-based module and using compositional features for the model. Due to this, the method is extremely fast and computationally inexpensive while achieving comparable performance with the more complex method—DM3Loc. The time taken by MRSLpred for predicting the location of 500 mRNA sequences is less than 1 min, whereas for the same dataset, DM3Loc takes approximately 32 min.

Sequence-based classification has its drawbacks as it tends to lose structural information, which is also known to play an essential role in subcellular location. However, in order to use structure-based features for prediction, more computational power may be required. Furthermore, the dataset used in this study had an inherent class imbalance, which makes the model biased to classes represented in larger numbers (the nucleus and exosome). In the future version of our tool, we expect to obtain a more balanced dataset where all locations have equal representation.

It is worth mentioning that we could have achieved better performance with deep learning methods. We tried to implement a CNN classifier based on one-hot encoded sequences, but the maximum average AUROC achieved for all the locations was 0.584 on the validation dataset. The CNN classifier performed very well on the training dataset (average AUROC = 0.837) but failed miserably on the validation dataset.

## 5 Conclusion

In this tool, we managed to develop a multi-label subcellular localization prediction tool that can accurately identify all the possible subcellular locations that an mRNA could move to. Our final model was based on an XGBoost multi-label classifier that also deploys a motif module to improve our prediction. We managed to achieve an AUROC of 0.708–0.816 (average: 0.742) and an MCC of 0.073–0.319 (average: 0.232). Due to the simpler architecture of our tool, it is extremely fast, and the standalone can be run on very minimal computational power. We believe that this will prove to be a valuable tool for biologists who work with mRNA localization. MRSLpred is available online at https://webs.iiitd.edu.in/raghava/mrslpred/, and its standalone can be downloaded from https://webs.iiitd.edu.in/raghava/mrslpred/standalone.php. The standalone is also available on GitHub and is accessible at https://github.com/raghavagps/mrslpred.

## Data Availability

Publicly available datasets were analyzed in this study. These data can be found online at: https://webs.iiitd.edu.in/raghava/mrslpred/downloads.php.
